# Embelin Restores Carbapenem Efficacy against NDM-1-Positive Pathogens

**DOI:** 10.3389/fmicb.2018.00071

**Published:** 2018-01-25

**Authors:** Nian-Zhi Ning, Xiong Liu, Fanghong Chen, Peng Zhou, Lihong Hu, Jian Huang, Zhan Li, Jie Huang, Tao Li, Hui Wang

**Affiliations:** ^1^State Key Laboratory of Pathogens and Biosecurity, Beijing Institute of Microbiology and Epidemiology, Beijing, China; ^2^Key Laboratory for NeuroInformation of Ministry of Education, University of Electronic Science and Technology of China, Chengdu, China; ^3^Center for Information in Biomedicine, University of Electronic Science and Technology of China, Chengdu, China; ^4^Shanghai Institute of Materia Medica, Chinese Academy of Sciences, Shanghai, China

**Keywords:** embelin, carbapenem, NDM-1, carbapenemase, inhibitor

## Abstract

The emergence and spread of carbapenemase in Gram-negative pathogens poses an enormous threat to global public health. New Delhi metallo-β-lactamase-1 (NDM-1) inactivates nearly every class of β-lactam antibiotics, including carbapenem; however, there is no clinically useful NDM-1 inhibitor. Embelin, an important ingredient in traditional herbal medicine, has anti-tumor effects. The current study is the first to discover and examine the inhibitory activity of embelin against β-lactamase NDM-1. The IC_50_ of embelin was 2.1 ± 0.2 μM when tested against NDM-1 carbapenemase. Most regions of the embelin molecule were buried within NDM-1’s active site, and the hydroxyl group of embelin interacted directly with the metal ion Zn^2+^, as shown by molecular dynamic simulation. Systematic analysis of the antibacterial activities of embelin and antibiotics demonstrated that embelin restored meropenem activity against a panel of NDM-positive pathogens, such as *Escherichia coli, Klebsiella pneumoniae*, and *Acinetobacter baumannii*. Based on these results, embelin could be a promising carbapenem adjuvant candidate against NDM-1-producing bacterial strains.

## Introduction

Carbapenems are antibiotics that are used for severe and difficult-to-treat infections caused by Gram-negative bacteria. They have broad spectrum antibacterial activity and are stable in the presence of most β-lactamases (Nordmann et al., 2011). Therefore, the emergence and spread of carbapenem-resistant Gram-negative pathogens pose an enormous threat to global public health (Laxminarayan et al., 2013). The acquisition of metallo-β-lactamases (MBLs), such as NDM-1, is one of the ways that Gram-negative pathogens become resistant to carbapenems, thus threatening the usefulness of penicillin, cephalosporin and carbapenem when treating these infections (King and Strynadka, 2013).

New Delhi metallo-β-lactamase 1 (NDM-1) is a relatively recent and emerging concern among the heterogeneous group of carbapenemases. NDM-1 was first described in *Klebsiella pneumoniae* and *Escherichia coli*, which were isolated from an Indian patient in Sweden in 2008 (Yong et al., 2009). In recent years, there has been rapid and widespread dissemination of NDM-1-positive strains in a number of countries (Kumarasamy et al., 2010). In addition, NDM-1 has been identified in a variety of Enterobacteriaceae and *Acinetobacter* and *Pseudomonas* species. The *bla*_NDM-1_ gene has been reported to be carried by different plasmid types, and it is likely that *bla*_NDM-1_ mobilization and insertion into various related organisms originates with these plasmids (Johnson and Woodford, 2013; Jin et al., 2015). The NDM-1 enzyme exhibits high hydrolytic efficiency against most clinically used β-lactam antimicrobials (Poirel et al., 2011; Thomas et al., 2011; Li et al., 2013). To make matters worse, frequently used inhibitors, such as clavulanic acid, sulbactam and tazobactam, do not inhibit NDM-1 activity. To date, no clinical MBL-inhibitor has been found that can reverse resistance and re-sensitize Gram-negative pathogens to carbapenems (Guo and Ma, 2014).

Embelin, a benzoquinone-derivative isolated from *Embelia ribes* has been identified as a potent inhibitor of X-linked inhibitor of apoptosis protein (XIAP) (Nikolovska-Coleska et al., 2004; Ahn et al., 2007). In addition, one study reported that embelin has potential antibacterial activity (Chitra et al., 2003). Our study found that embelin is a potent inhibitor against the hydrolysis activity of NDM-1. The aim of the current study was to evaluate the inhibition of the NDM-1 enzyme by embelin and to determine the efficacy of various β-lactam/embelin combinations against pathogens that harbor NDM-1.

## Materials and Methods

### Antibacterial Agents and Bacterial Strains

Embelin (purity > 98%), meropenem, biapenem, cefepime ampicillin, cefradine, clavulanic acid, sulbactam, and tazobactam were purchased from Sigma Co. (United States). ATCC BAA-2146 was purchased from the American Type Culture Collection.

### Enzyme Activity-Based Screen

NDM-1 was expressed and purified as previously described (Li et al., 2013). The purity of NDM-1 assessed by SDS–PAGE and was > 95%. The purified NDM-1 was used to determine the kinetic parameter, Km as described previously and was in agreement with previously published values (Li et al., 2013). Inhibition of enzyme activity was determined by comparing the initial hydrolysis rates of meropenem between NDM-1 and a mixture of NDM-1 with different compounds. Hundreds of natural product extracts and chemicals, including embelin, curcumine and resveratrol were screened for inhibitory activity. Reactions were performed at 30°C in a total volume of 200 μL 4-(2-hydroxyethyl)-1-piperazineethanesulfonic acid (HEPES) (50 mM, pH 7.5) buffer containing 50 μM ZnSO_4_. The initial hydrolysis rates of meropenem were measured using a SynergyHT spectrophotometer (Biotek Corp., United States) at a wavelength of 297 nm (Li et al., 2013). The same assay was performed with VIM-1 and IMP-1.

### K_i_ and IC_50_ Assays

Prior to testing with 0.5 mM meropenem, the NDM-1 (0.5 μg/ml) enzyme was incubated with various concentrations of embelin in 10 mM HEPES (pH 7.5) buffers at 30°C for 10 min. The IC_50_ was defined as the concentration of inhibitor needed to reduce the initial rate of hydrolysis of meropenem by 50% (Li et al., 2013), and the K_i_ (inhibitory constant) was determined using GraphPad Prism software. Experiments were performed independently at least three times. The same methods were used to measure the IC_50_ and K_i_ of VIM-1 and IMP-1.

### *In Vitro* Susceptibility Tests

*In vitro* susceptibility tests were performed using the Mueller–Hinton broth microdilution technique as recommended by the (Clinical and Laboratory Standards Institute [CLSI], 2012) Briefly, the selected antibiotics were diluted from 4096 to 0.125 μg/ml by the broth two-fold dilution method. Different concentrations of embelin (from 1 to 64 μg/ml) were added in combination with β-lactam antibiotics. The inoculum (final size, 5 × 10^5^ CFU/ml) was prepared by the direct colony suspension method.

### FIC Index Determination

Fractional inhibitory concentration (FIC) values were determined using standard methods (Pillai et al., 2005). Checkerboards were set up with 8 concentrations of each meropenem and embelin in serial one-half dilutions. The minimal inhibitory concentration (MIC) for each compound was determined by the broth microdilution technique as recommended by (Clinical and Laboratory Standards Institute [CLSI], 2012). The FIC for each compound was calculated as the compound in the presence of co-compound concentration for a well showing no growth, divided by the MIC for that compound (King et al., 2014). The FIC index was taken as the sum of the two FICs. Experiments were performed three times and the means were used for calculation.

### Clinical Isolates Screening

Various concentrations of embelin (from 2 to 16 μg/ml) were tested in combination with 2 μg/ml of meropenem, which is the EUCAST breakpoint for resistance (King et al., 2014). The synergistic properties of the two compounds were tested in microtiter plates. *Escherichia coli* ATCC 25922 was used as the control in all plates. Overall, 46 NDM-1-haboring clinical isolates (Enterobacteriaceae and Acinetobacter spp.), including 17 *E. coli*, 15 *K. pneumoniae* and 14 *A. baumannii*, were tested. After an 18-h incubation at 37°C, the plates were read. All strains were used to examine reproducibility and no deviation was shown.

### Molecular Docking

The flexible molecular docking method AutoDock (Goodsell and Olson, 1990) was used to analyze the intermolecular interaction between the NDM-1 protein and the small-molecule ligand, embelin. In the protocol, the high-resolution crystal structure of NDM-1 complexed with its potent competitive inhibitor L-captopril was used as a template to perform the docking (Gonzalez et al., 2012; King et al., 2012); the structure was retrieved from the PDB database (Berman et al., 2000) under the accession code 4EXS. The binding site for the docking calculations was defined as those residues that could directly contact the co-crystallized L-captopril. The binding site contained 11 amino acid residues (Met67, Val73, Trp93, His120, His122, Asp124, His189, Cys208, Gly219, Asn220 and His250) and two zinc ions. To prepare the site for docking, a rectangular grid was defined that covered the binding-site residues and zinc ions. All water molecules, as well as the cocrystallized L-captopril, were removed from the crystal structure system, while the coordinated Zn^2+^ was kept in NDM-1’s active site due to its important role in stabilizing the NDM-1–substrate/ligand interaction. The docking exploration of embelin’s binding conformations was performed in the rectangular gird. Subsequently, Gasteiger and Kollman united atomic charges were assigned for the embelin ligand and NDM-1 protein, respectively. The protein was also added with polar hydrogen atom. A grid was set to accommodate the active-site region with a 0.375 Å span. The torsion and rotatable bonds in the ligands were defined, and the non-polar hydrogens and partial atomic charges were added to the bonded carbon atoms (Wang et al., 2017). The docking was carried out using the AutoDock Vina program (Trott and Olson, 2010) to evaluate ligand binding energies over the conformational search space using the Lamarckian genetic algorithm.

### Data Availability

No datasets were generated or analyzed in the current study.

## Results

### Embelin Potently Inhibits NDM-1 Enzyme Activity

**FIGURE 1:**
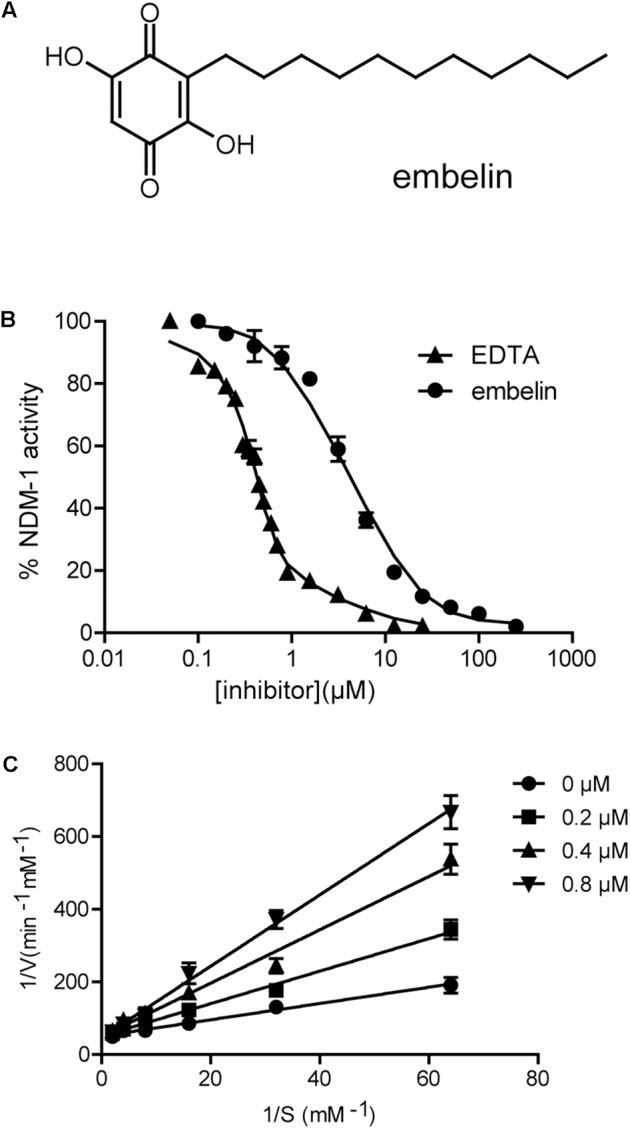
Embelin inactivates the enzyme activity of NDM-1. **(A)** Chemical structure of embelin. **(B)** Determination of the IC_50_ of embelin against NDM-1with 0.5 mM meropenem as a reporter substrate, EDTA was used as a control. The IC_50_ value is approximately 2.1 ± 0.2 μM. **(C)** Determination of the K_i_ of embelin against NDM-1with meropenem as reporter substrate.

An enzyme activity-based screening, using our in-house collection of natural product extracts and chemicals was initiated in order to find a new NDM-1 inhibitor. Three compounds (20 μM), embelin (**Figure 1A**), curcumine (**Supplementary Figure S1A**) and resveratrol (**Supplementary Figure S1B**), showed more than 20% inhibition. Among these three compounds, embelin inhibited NDM-1 by more than 50%. Dose-dependent analyses further revealed that embelin inhibited NDM-1 at an IC_50_ value of 2.1 ± 0.2 μM and K_i_ value of 0.19 ± 0.02 μM when meropenem was the substrate (**Figures 1B,C**). The IC_50_ value of embelin with imipenem as a substrate was 2.3 ± 0.4 μM, similar with that with meropenem (**Supplementary Figure S2**). The inhibitor activity of embelin against other metallo-beta-lactamases, such as VIM and IMP was also determined. The IC_50_ was approximately 200 μM against VIM-1 and 100 μM against IMP-1 with 0.5 mM meropenem as a substrate. These results suggest that embelin is a potent inhibitor of NDM-1, but a relatively weak inhibitor of VIM-1 and IMP-1.

### Molecular Dynamic Study of the NDM-1/Embelin Complex

**FIGURE 2:**
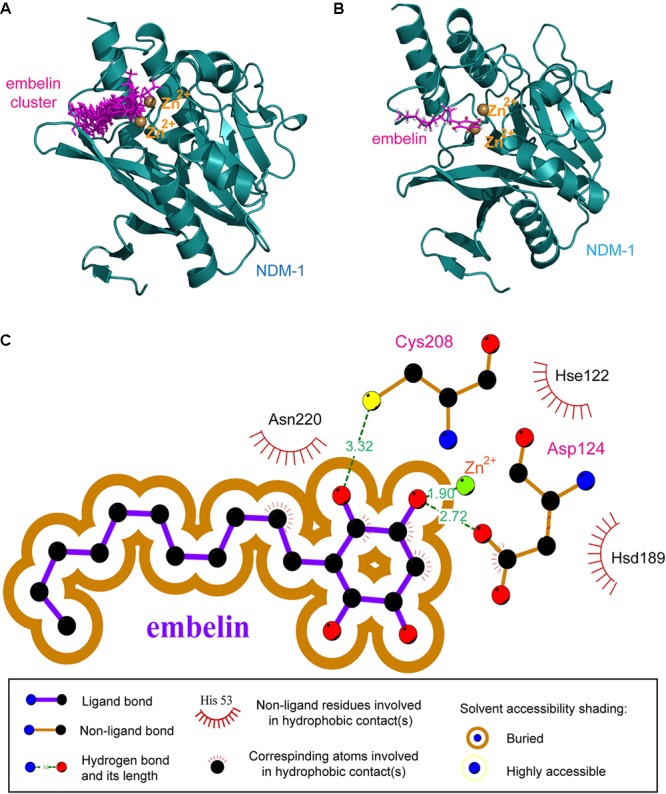
The resulting NDM-1–embelin binding mode resulting from molecular docking. **(A)** The 10 best embelin conformations positioned within the NDM-1 active site. **(B)** The average conformation of the 10 best embelin conformations bound to NDM-1. **(C)** Schematic representation of non-bonded interactions at the docked NDM-1–embelin complex interface.

Molecular docking was done to fully search the huge conformation space of the embelin molecule within NDM-1’s active site. An empirical scoring function was then employed to evaluate the relative binding potency of generated ligand conformations to protein receptors. Hundreds of potential binding conformations of the embelin ligand to the NDM-1 active site were predicted using AutoDock, and the top-10 embelin binding conformations (with the highest theoretical scores) were superposed in the NDM-1 active site (**Figure 2A**).

The 10 best conformations exhibited very similar binding modes toward the NDM-1 protein, with only slight fluctuations at their side groups and moieties. Interestingly, the fatty hydrocarbon chain of the embelin molecule also highly consistent in different docking modes, although this chain is highly flexible and thus may have significant swing when binding to a protein receptor. The average structures of the 10 best conformations were then clustered into one representative, which is shown in **Figure 2B**. As can be seen, the embelin molecule adopts its quinonyl moiety to interact with the catalytic pocket of the enzyme, while its fatty hydrocarbon chain points out of the pocket. In addition, the embelin is located near the di-Zn^2+^ cation, thus forming a strong coordination bond.

In order to elucidate the molecular mechanism involved in embelin binding to NDM-1, the non-bonded interactions at the complex interface of the NDM-1–embelin complex were determined using the LIGPLOT program (Wallace et al., 1995) and are shown in **Figure 2C**. Most regions of the embelin molecule are buried within NDM-1’s active site, suggesting that a wide van der Waals contact exists between NDM-1 and embelin, which should confer substantial stabilization for the complex architecture. In addition, although only limited hydrophobic forces are observed at the interface, there are two geometrically satisfactory hydrogen bonds that can be formed with residues Cys208 and Asp124; these are thought to constitute recognition specificity for the NDM-1–embelin binding. As might be expected, embelin can coordinate potently with the one of the di-Zn^2+^ through a hydroxyl group; this coordination bond was also found in the complex systems of NDM-1 with natural substrates and small-molecule inhibitors, suggesting the importance of the coordination bond in NDM-1–ligand recognition and interaction.

### Antibacterial Activity of β-lactam/Embelin Combinations against NDM-1 -Producing Strains

**FIGURE 3:**
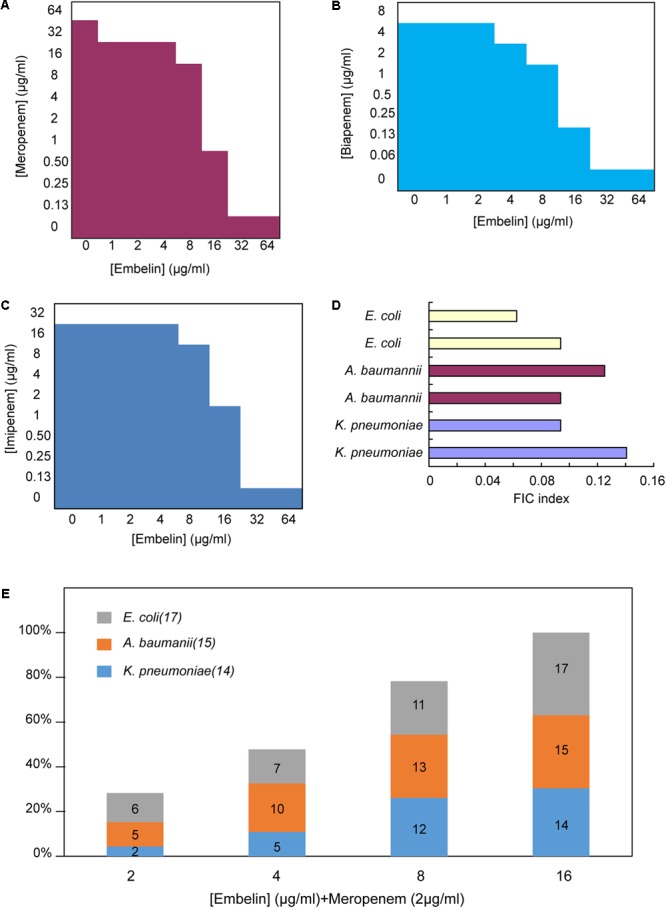
Embelin potentiates the activity of meropenem or biapenem against carbapenem-resistant pathogens **(A–C)**. Microdilution checkerboard analysis showing the combined effect of embelin and meropenem **(A)**, biapenem **(B)** and imipenem **(C)** selectively against *K. pneumoniae* ATCC BAA-2146 (MIC meropenem = 64 μg/ml, MIC biapenem = 8 μg/ml, MIC imipenem = 32 μg/ml)showing the average of three technical replicates. **(D)** FIC indices of embelin and meropenem against NDM-1 producing clinical isolates. **(E)** NDM-expressing Gram-negative pathogens were highly susceptible to the combination of meropenem (2 μg/ml) and embelin (from 2 to 16 μg/ml).

ATCC BAA-2146, a *K. pneumoniae* strain harboring the *bla*_NDM-1_ gene was tested for susceptibility to different β-lactam antibiotics. The *NDM-1* gene was verified using PCR. The selected antibiotics were diluted via serial twofold dilutions ranging from 4096 to 0.125 μg/ml. The strain was found to be highly resistant to the penicillins, cephalosporins and carbapenems tested. To detect inhibition activity, embelin, clavulanic acid, sulbactam or tazobactam was added to β-lactam antibiotics at different concentrations ranging from 1 to 64 μg/ml (**Figures 3A–C** and **Table 1**). The MIC values for the β-lactam antibiotics were not lowered by clavulanic acid, sulbactam or tazobactam (at 64 μg/ml) in the *K. pneumoniae* strain producing the NDM-1 enzyme. In contrast, embelin efficiently rescued the antibiotic activity of various β-lactam antibiotics. *In vitro* MICs against the NDM-1-producing strain (ATCC BAA-2146) were reduced 2- to 512-fold with embelin (**Figures 3A–C** and **Table 1**).

**Table 1:** Minimal inhibitory concentrations (MICs) of embelin in combination with different β-lactams against NDM-1-producing Bacteria.

Antimicorbials treated with embelin	MIC in μg/ml [with different concentrate embelin (μg/ml)]					
	0	1	2	4	8	16	32	64
Meropenem	64	32	32	32	16	1	0.125	0.125
Imipenem	32	32	32	32	16	2	0.125	0.125
Biapenem	8	8	8	4	2	0.25	0.0625	0.0625
Cefepime	256	64	64	64	32	32	32	8
Cefradine	4096	4096	4096	4096	4096	2048	1024	1024
Ceftazidime	2048	2048	2048	2048	1024	64	64	64
Ampicillin	>4096	>4096	>4096	>4096	>4096	>4096	>4096	4096

The antibacterial activity of embelin was analyzed to distinguish between NDM-1 inhibition and antibacterial activity. Embelin (128 μg/ml) alone did not inhibit the growth of ATCC BAA-2146. The MICs of various β-lactam antibiotics were very different from 4096 to 0.0625 μg/ml, when combined with an embelin concentration of 64 μg/ml. Ampicillin is a penam compound and has a high MIC (>4096 μg/ml) against ATCC BAA-2146. Only when the concentration of embelin reached 64 μg/ml, was the MIC of ampicillin reduced to 4096 μg/ml. When combined with embelin (64 μg/ml), the activity of cephem antibotics (cefradine, ceftazidime and cefepime) improved, but was not restored to susceptible levels. This may reflect a high efficiency of NDM-1 against amoxicillin and cephem antibotics, as the NDM-1 enzyme has been shown to exhibit high catalytic efficiencies for amoxicillin and cephem compound substrates (Thomas et al., 2011; Li et al., 2013). Compared with the other classes of antibotics, the MICs of carbapenems (meropenem, biapenem) were significantly decreased in the presence of embelin. When treated with 32 μg/ml embelin, the MICs of ATCC BAA-2146 against meropenem fell by up to 512-fold, 128-fold for biapenem and about 256-fold for imipenem. In the current study, the addition of embelin could reduce MIC values to below susceptibility breakpoints. Thus, the mixture of meropenem (or biapenem, or imipenem) with embelin could be as a candidate combination used to treat NDM-1-producing strains.

Systematic titration of concentrations of embelin and meropenem against NDM-1 positive clinical bacterial strains showed that embelin restored meropenem activity consistent with NDM inhibition. FIC index values were determined to be 0.05 – 0.15 for a panel of 6 clinical NDM-1 positive isolates (2 *E. coli*, 2 *K. pneumoniae* and 2 *A*. *baumannii*) tested against a combination of meropenem and embelin (FIC values of ≤ 0.5 are defined as synergistic (Pillai et al., 2005) (**Figure 3D**). Different concentrations of embelin (from 2 to 16 μg/ml) combined with meropenem were further investigated using 46 NDM-1 positive clinical isolates (17 *E. coli*, 15 *K. pneumoniae* and 14 *A. baumannii*, **Figure 3E**). Embelin at 2, 4, 8, and 16 μg/ml restored meropenem sensitivity (2 mg/ml) in 28.3, 47.8, 78.3, and 100% of NDM positive isolates, respectively. An additional experiment was performed to evaluate the inhibitory activity of embelin alone, using the 46 isolates. Embelin (64 μg/ml) alone did not significantly inhibit activity against any of the strains of *E. coli, K. pneumoniae*, or *A. baumannii* tested. These results demonstrate that embelin could restore carbapenem efficacy against NDM-1-positive pathogens.

## Discussion

A previous study reported that NDM-1-positive organisms tended to be highly resistant to all β-lactam antibiotics tested (Kumarasamy et al., 2010). The emergence and fast spread of NDM-1 in hospital acquired pathogens make the quest for inhibitors of these enzymes an urgent clinical need. Several NDM-1 inhibitors have been reported, and most of them are based on Zn-dependent inhibition (Rotondo and Wright, 2017). Captopril is a well-known hypertension drug, due to its ability to chelate zinc ions through a free thiol, both D-and L-captopril have been reported as inhibitors of NDM-1, with IC_50_ values of ∼8 μM and ∼200 μM, respectively (Guo et al., 2011; King et al., 2012). Using similar metal chelation mechanism, many other examples of thiol-containing compounds have been reported, however, adverse effects associated with these kind of compounds may present a weakness for captopril and its derivatives (Kitamura et al., 1990). Aspergillomarasmine A is a natural fungal product and inhibits NDM-1 (IC_50_ = 4.0 μM) through a metal sequestration mechanism, which can effectively rescues meropenem activity in a mouse systemic infection model (King et al., 2014). Boronic acid containing compounds are found to show outstanding inhibition activity against NDM-1 (IC_50_ = 4 nM) so far (Brem et al., 2016). But, no inhibitor against NDM-1 has been in human clinical trials, showing the importance of identifying new molecules.

Embelin is a promising agent for treating infections caused by NDM-1-producing pathogens via protecting β-lactam antibiotics from hydrolysis. Data from the current study showed that embelin is a potent inhibitor of NDM-1 enzymes, with an IC_50_ value of 2.1 ± 0.2 μM and a K_i_ value of 0.19 ± 0.02 μM. In addition, embelin rescued the antibiotic activity of meropenem, imipenem and biapenem when used to treat pathogens producing the NDM-1 enzyme.

In published reports (Poojari et al., 2010; Poojari, 2014), acute toxicity studies in mice treated with 50 or 100 mg/kg oral dose of embelin showed no significant change in body weight, mortality or any apparent toxic effects, indicating that embelin has a good safety profile. However, some potential side effects and toxicity resulting from embelin have been reported. Different suppliers, such as Cayman Chemicals and Santa Cruz Biotechnology, catalog embelin as “suspected of damaging fertility or the unborn child” and classify it as “toxic for reproduction, category 2.” Thus, the embelin molecule could be as a potential “lead compound,” from which other “safer” molecules can be derived and further developed to new carbapenem adjuvants against NDM-1-producing bacterial strains.

## Author Contributions

TL and HW designed the research study. LH provided the compounds. PZ and JiaH performed the bioinformatic analysis. N-ZN, FC, XL, ZL, and JieH performed the experiment and data analysis. N-ZN, XL, and TL wrote the manuscript.

## Conflict of Interest Statement

The authors declare that the research was conducted in the absence of any commercial or financial relationships that could be construed as a potential conflict of interest.

## Supplementary Material

FIGURE S1 Chemical structure of curcumine. **(B)** Chemical structure of resveratrol.

FIGURE S2 Determination of the IC_50_ of embelin against NDM-1 with 0.5 mM Imipenem as reporter substrate. The IC_50_ value is approximately 2.3 ± 0.4 μM.

## References

[B1] Ahn K. S. Sethi G. Aggarwal B. B. (2007). Embelin, an inhibitor of X chromosome-linked inhibitor-of-apoptosis protein, blocks nuclear factor-kappaB (NF-kappaB) signaling pathway leading to suppression of NF-kappaB-regulated antiapoptotic and metastatic gene products. *Mol. Pharmacol.* 71 209–219. 10.1124/mol.106.028787 17028156

[B2] Berman H. M. Westbrook J. Feng Z. Gilliland G. Bhat T. N. Weissig H. (2000). The protein data bank. *Nucleic Acids Res.* 28 235–242. 10.1093/nar/28.1.235 10592235 PMC102472

[B3] Brem J. Cain R. Cahill S. Mcdonough M. A. Clifton I. J. Jimenez-Castellanos J. C. (2016). Structural basis of metallo-beta-lactamase, serine-beta-lactamase and penicillin-binding protein inhibition by cyclic boronates. *Nat. Commun.* 7:12406. 10.1038/ncomms12406 27499424 PMC4979060

[B4] Chitra M. Devi C. S. Sukumar E. (2003). Antibacterial activity of embelin. *Fitoterapia* 74 401–403. 10.1016/S0367-326X(03)00066-2 12781816

[B5] Clinical and Laboratory Standards Institute [CLSI] (2012). *Methods for Dilution Antimicrobial Susceptibility Tests for Bacteria that Grow Aerobically; Approved Standard-Ninth Edition M07-A9.* Wayne, PA: CLSI.

[B6] Gonzalez J. M. Meini M. R. Tomatis P. E. Medrano Martin F. J. Cricco J. A. Vila A. J. (2012). Metallo-beta-lactamases withstand low Zn(II) conditions by tuning metal-ligand interactions. *Nat. Chem. Biol.* 8 698–700. 10.1038/nchembio.1005 22729148 PMC3470787

[B7] Goodsell D. S. Olson A. J. (1990). Automated docking of substrates to proteins by simulated annealing. *Proteins* 8 195–202. 10.1002/prot.340080302 2281083

[B8] Guo Y. Wang J. Niu G. Shui W. Sun Y. Zhou H. (2011). A structural view of the antibiotic degradation enzyme NDM-1 from a superbug. *Protein Cell* 2 384–394. 10.1007/s13238-011-1055-9 21637961 PMC4875342

[B9] Guo Z. Ma S. (2014). Recent advances in the discovery of metallo-beta-lactamase inhibitors for beta-lactam antibiotic-resistant reversing agents. *Curr. Drug Targets* 15 689–702. 10.2174/1389450115666140326094504 24666360

[B10] Jin Y. Shao C. Li J. Fan H. Bai Y. Wang Y. (2015). Outbreak of multidrug resistant NDM-1-producing *Klebsiella pneumoniae* from a neonatal unit in Shandong Province, China. *PLOS ONE* 10:e0119571. 10.1371/journal.pone.0119571 25799421 PMC4370709

[B11] Johnson A. P. Woodford N. (2013). Global spread of antibiotic resistance: the example of New Delhi metallo-beta-lactamase (NDM)-mediated carbapenem resistance. *J. Med. Microbiol.* 62 499–513. 10.1099/jmm.0.052555-0 23329317

[B12] King A. M. Reid-Yu S. A. Wang W. King D. T. De Pascale G. Strynadka N. C. (2014). Aspergillomarasmine A overcomes metallo-beta-lactamase antibiotic resistance. *Nature* 510 503–506. 10.1038/nature13445 24965651 PMC4981499

[B13] King D. T. Strynadka N. C. (2013). Targeting metallo-beta-lactamase enzymes in antibiotic resistance. *Future Med. Chem.* 5 1243–1263. 10.4155/fmc.13.55 23859206

[B14] King D. T. Worrall L. J. Gruninger R. Strynadka N. C. (2012). New Delhi metallo-beta-lactamase: structural insights into beta-lactam recognition and inhibition. *J. Am. Chem. Soc.* 134 11362–11365. 10.1021/ja303579d 22713171

[B15] Kitamura K. Aihara M. Osawa J. Naito S. Ikezawa Z. (1990). Sulfhydryl drug-induced eruption: a clinical and histological study. *J. Dermatol.* 17 44–51. 10.1111/j.1346-8138.1990.tb01608.x 2139441

[B16] Kumarasamy K. K. Toleman M. A. Walsh T. R. Bagaria J. Butt F. Balakrishnan R. (2010). Emergence of a new antibiotic resistance mechanism in India, Pakistan, and the UK: a molecular, biological, and epidemiological study. *Lancet Infect. Dis.* 10 597–602. 10.1016/S1473-3099(10)70143-2 20705517 PMC2933358

[B17] Laxminarayan R. Duse A. Wattal C. Zaidi A. K. Wertheim H. F. Sumpradit N. (2013). Antibiotic resistance-the need for global solutions. *Lancet Infect. Dis.* 13 1057–1098. 10.1016/S1473-3099(13)70318-9 24252483

[B18] Li T. Wang Q. Chen F. Li X. Luo S. Fang H. (2013). Biochemical characteristics of New Delhi metallo-beta-lactamase-1 show unexpected difference to other MBLs. *PLOS ONE* 8:e61914. 10.1371/journal.pone.0061914 23593503 PMC3625156

[B19] Nikolovska-Coleska Z. Xu L. Hu Z. Tomita Y. Li P. Roller P. P. (2004). Discovery of embelin as a cell-permeable, small-molecular weight inhibitor of XIAP through structure-based computational screening of a traditional herbal medicine three-dimensional structure database. *J. Med. Chem.* 47 2430–2440. 10.1021/jm030420+ 15115387

[B20] Nordmann P. Naas T. Poirel L. (2011). Global spread of Carbapenemase-producing Enterobacteriaceae. *Emerg. Infect. Dis.* 17 1791–1798. 10.3201/eid1710.110655 22000347 PMC3310682

[B21] Pillai S. K. Moellering R. C. J. Eliopoulos G. M. (2005). *Antibiotics in Laboratory Medicine* ed. Lorian V. (Philadelphia, PA: Williams & Wilkins) 365–440.

[B22] Poirel L. Dortet L. Bernabeu S. Nordmann P. (2011). Genetic features of blaNDM-1-positive Enterobacteriaceae. *Antimicrob. Agents Chemother.* 55 5403–5407. 10.1128/AAC.00585-11 21859933 PMC3195013

[B23] Poojari R. (2014). Embelin - a drug of antiquity: shifting the paradigm towards modern medicine. *Expert Opin. Investig. Drugs* 23 427–444. 10.1517/13543784.2014.867016 24397264

[B24] Poojari R. Gupta S. Maru G. Khade B. Bhagwat S. (2010). Chemopreventive and hepatoprotective effects of embelin on N-nitrosodiethylamine and carbon tetrachloride induced preneoplasia and toxicity in rat liver. *Asian Pac. J. Cancer Prev.* 11 1015–1020. 21133617

[B25] Rotondo C. M. Wright G. D. (2017). Inhibitors of metallo-beta-lactamases. *Curr. Opin. Microbiol.* 39 96–105. 10.1016/j.mib.2017.10.026 29154026

[B26] Thomas P. W. Zheng M. Wu S. Guo H. Liu D. Xu D. (2011). Characterization of purified New Delhi metallo-beta-lactamase-1. *Biochemistry* 50 10102–10113. 10.1021/bi201449r 22029287

[B27] Trott O. Olson A. J. (2010). AutoDock Vina: improving the speed and accuracy of docking with a new scoring function, efficient optimization, and multithreading. *J. Comput. Chem.* 31 455–461. 10.1002/jcc.21334 19499576 PMC3041641

[B28] Wallace A. C. Laskowski R. A. Thornton J. M. (1995). LIGPLOT: a program to generate schematic diagrams of protein-ligand interactions. *Protein Eng.* 8 127–134. 10.1093/protein/8.2.127 7630882

[B29] Wang Y. Yang C. Xue W. Zhang T. Liu X. Ju J. (2017). Selection and characterization of alanine racemase inhibitors against *Aeromonas hydrophila*. *BMC Microbiol.* 17:122. 10.1186/s12866-017-1010-x 28545531 PMC5445283

[B30] Yong D. Toleman M. A. Giske C. G. Cho H. S. Sundman K. Lee K. (2009). Characterization of a new metallo-beta-lactamase gene, bla(NDM-1), and a novel erythromycin esterase gene carried on a unique genetic structure in *Klebsiella pneumoniae* sequence type 14 from India. *Antimicrob. Agents Chemother.* 53 5046–5054. 10.1128/AAC.00774-09 19770275 PMC2786356

