# Supplementary figures and images for: Embelin Restores Carbapenem Efficacy against NDM-1-Positive Pathogens
Source: Front Microbiol. 2018 Jan 25;9:71. doi: 10.3389/fmicb.2018.00071 (PMC5789148; doi:10.3389/fmicb.2018.00071)

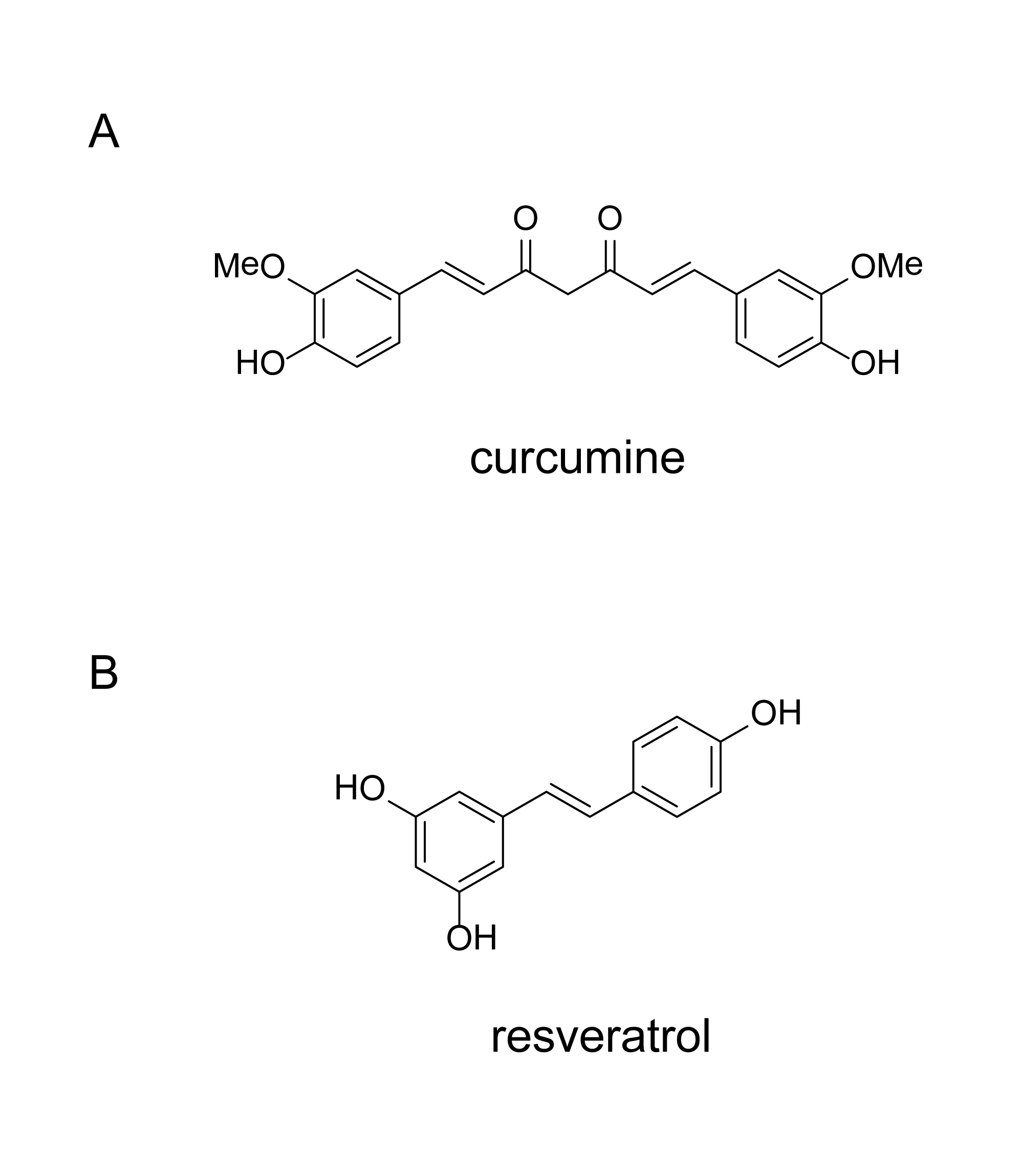

Supplement: FIGURE S1 — Chemical structure of curcumine. (B) Chemical structure of resveratrol. [file Image_1.TIF]

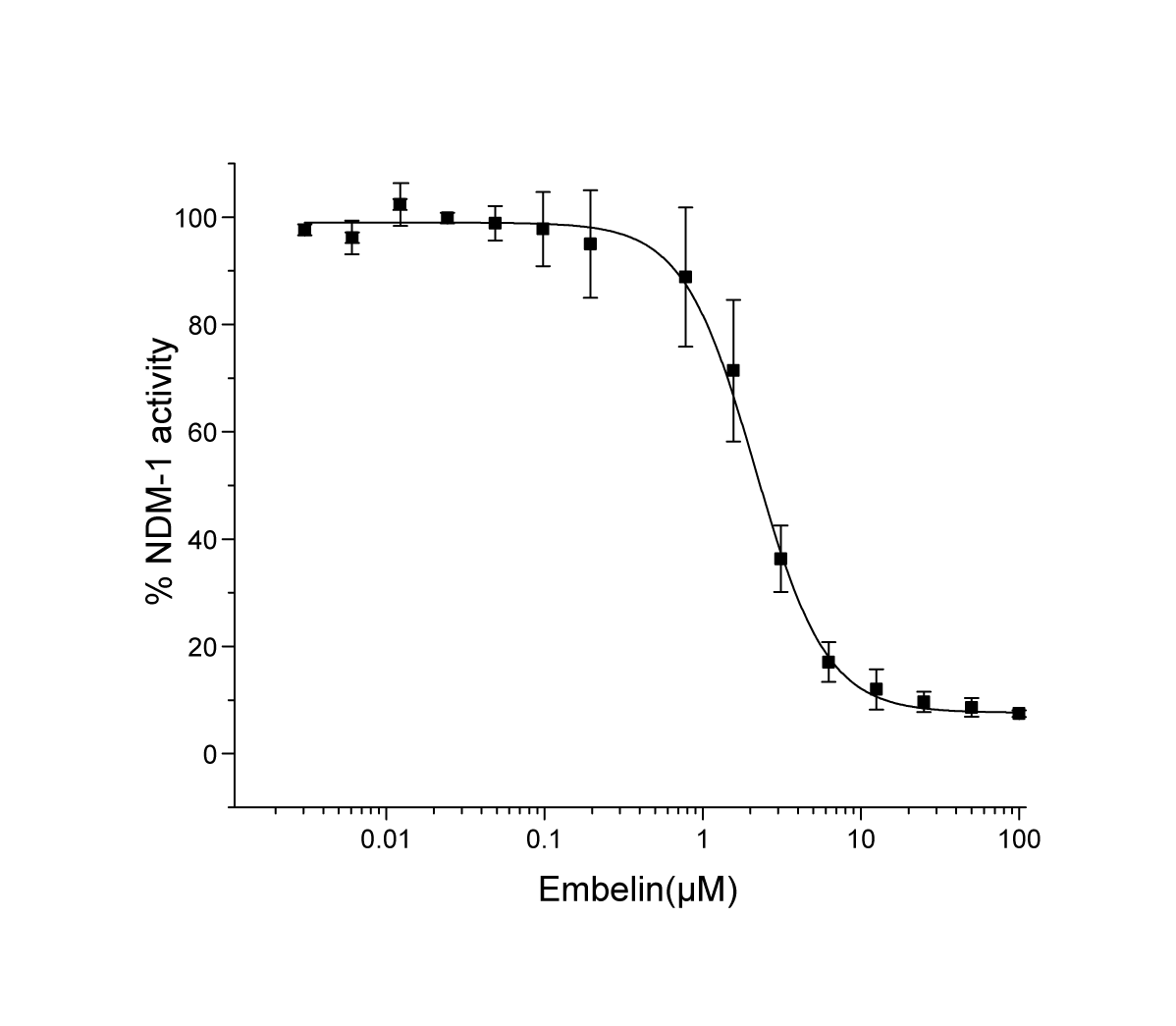

Supplement: FIGURE S2 — Determination of the IC50 of embelin against NDM-1 with 0.5 mM Imipenem as reporter substrate. The IC50 value is approximately 2.3 ± 0.4 μM. [file Image_2.TIF]
